# Meta-analysis of lean and obese RNA-seq datasets to identify genes targeting obesity

**DOI:** 10.6026/97320630019331

**Published:** 2023-03-31

**Authors:** Lavanya Prabhakar, Dicky John Davis G

**Affiliations:** 1 Department of Bioinformatics, Faculty of Engineering and Technology, Sri Ramachandra Institute of Higher Education and Research (DU), Chennai, Tamil Nadu - 600116, India

**Keywords:** Differential gene expression (DEG), Galaxy server, Gene enrichment analysis and MCODE

## Abstract

Obesity is a global crisis leading to several metabolic disorders. Modernization and technology innovation has been easier for next generation sequencing using open-source online software galaxy, which allows the users to share their data and workflow mapping in an effortless manner. This study is to identify candidate genes for obesity by performing differential expression of genes. RNA-Seq analysis was performed for six different datasets retrieved from GEO database. 258 datasets from obese patients and 55 datasets from lean patients were analysed for differentially expressed genes (DEGs). DEGs analysis showed 1971 upregulated genes and 615 downregulated genes with log2FC count ≥ 2.5 and p-value < 0.05. The Gene enrichment analysis performed using Gene Ontology resource highlighted pathways associated to obesity such as cholesterol metabolism, Fat digestion and absorption and glycerolipid metabolism. Using string database protein-protein interactions network was built and the network clusters were visualized using Cytoscape software. The protein-protein interactions of the upregulated and downregulated genes were mapped to form a network, wherein PNLIP (Pancreatic lipase) and FTO (Fat mass and obesity associated protein) gene clusters were visualized as densely connected clusters in MCODE. PNLIP and FTO with its associated genes were identified as candidate genes for targeting obesity.

## Abbreviations:

PNLIP- Pancreatic Lipase; FTO- Fat mass and obesity associated protein; DEG- Differential gene expression; KEGG- Kyoto Encyclopedia of Genes and Genomes; PPI- Protein-protein interactions; MCODE- Molecular Complex Detection.

## Background:

Obesity is increasing at an alarming rate leading to various metabolic diseases [1]. The identification of candidate gene for obesity is highly important for treating this global epidemic crisis [2]. Modernization and technology innovation created novel sequencing technologies in genome sequencing whereby large DNA fragments were detected using Next Generation sequencing technique (NGS) [3]. In recent years, RNA sequencing has been widely exploited to continuously monitor the changes in cellular transcriptome [4]. The objective of RNA-Seq is to create profiling of gene expressions by identification of genes or their corresponding molecular pathways and understanding the differentially expressed genes among two or more biological conditions using galaxy platform. The dataset for obesity is imported from public databases to identify differentially expressed genes (DEG) involved in obesity.

## Materials and Methods:

Next generation RNA sequencing samples were retrived from NCBI GEO Database from 5 different studies namely GSE152991 [5], GSE132831 [6], GSE86430 [7], GSE148892 [8], GSE161042 [9] and GSE137631[10] (Table 1). A total of 313 samples of which, 258 samples were from obese patients and 55 samples were from lean patients. The datasets were imported into the Galaxy Server (https://usegalaxy.org.au/) using the tool Faster Download and Extract Reads in FASTQ format from NCBI SRA (Galaxy Version 2.11.0 + galaxy0).The read quality check was performed by using the tool FastQC Read Quality reports (Galaxy Version 0.73 + galaxy0). Trimmomatic, a flexible read trimming tool for Illumina NGS data (Galaxy Version 0.36.6) [11] was run with default parameters and phred quality score. The consolidated report was generated using MultiQC aggregate results from bioinformatics analyses into a single report (Galaxy Version 1.11 + galaxy0) [12]. Sequence Mapping and Alignment was performed using HISAT2, a fast and sensitive alignment program (Galaxy Version 2.2.1 + galaxy1) [13]. The RNA sequence reads were mapped to reference human genome version hg38. FeatureCounts was used to measure gene expression in RNA-Seq experiments from BAM files (Galaxy Version 2.0.1 + galaxy2) . Annotations for gene regions were provided in the GTF format. Differential expression gene analysis was performed on two factors namely, Obese VS lean patients with limma-voom (Galaxy Version 3.50.1+galaxy0) [14].

The differentially expressed upregulated and downregulated genes were submitted to online tool g:GOSt to perform functional enrichment analysis. The DEGs were subjected to Gene ontology (GO) resources (http://www.geneontology.org/). It maps genes to known functional information sources and detects statistically significantly enriched terms. The GO analysis having terms under the three categories such as cellular component (CC), molecular function (MF) and biological process (BP) are completed. The cut-off value for a significant GO term and pathway was set to p-value< 0.05 and log2FC count ≥ 2.5. To further analyses the potential pathway of the overlapping DEGs, gene ontology resources integrate pathways from Kyoto Encyclopedia of Genes and Genomes (KEGG) [15] was used to perform pathway enrichment analysis.

The protein-protein interactions (PPI) network of the obesity genes associated were constructed with the help of the online Search Tool for the Retrieval of Interacting Genes (STRING) database [16]. Cytoscape (version 3.9.1) [17], was used for the visualization of the PPI by importing the tsv file of the STRING database. Cytoscape helps to organize the imported network as a graph by representing the molecular species in the form of nodes and edges, where each node represented a protein product of single-gene and edges represented the protein-protein association. The Molecular Complex Detection (MCODE) [18] plugin of the Cytoscape app was used to identify the densely connected regions/clusters in the PPI network. The top ranked gene clusters of the interactive network were extracted according to their scores.

## Results

**Figure 1:**
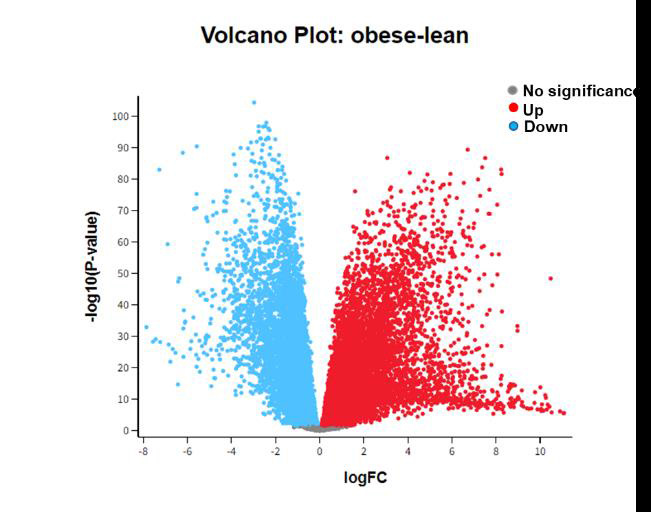
Volcano plot of differentially expressed genes.

To preliminarily understand the mechanism contributing to the obesity, 313 patients (258 obese patients and 55 lean patients) were selected for subsequent analysis. The differentially expressed genes of obese VS lean samples from limma-voom were examined using volcano plot. The volcano plot represents the expressed fold change of genes in obese vs lean samples were plotted against the degree of statistical significance in differential expression (Figure 1).

**Figure 2:**
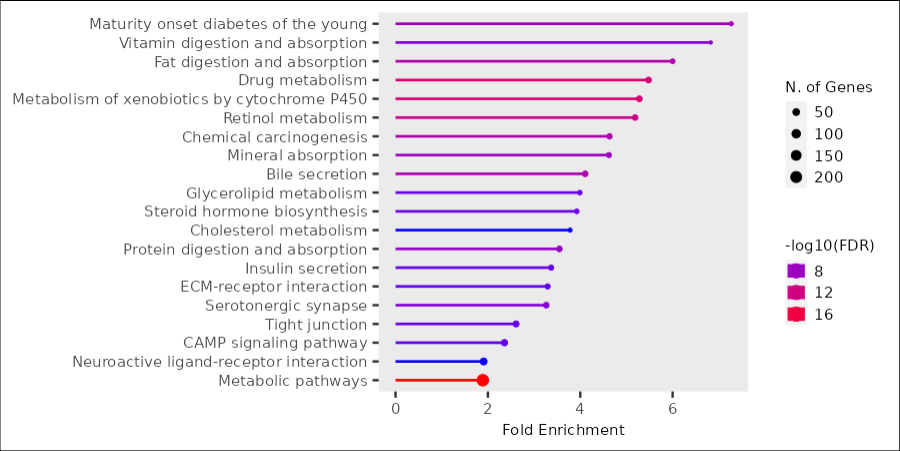
The fold enrichment pathway for upregulated genes

**Figure 3:**
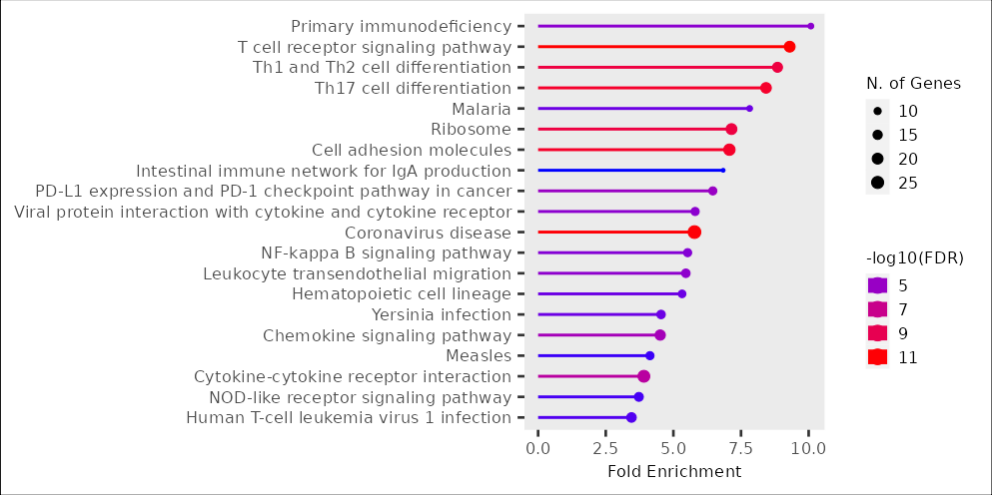
The fold enrichment pathway for downregulated genes

A total of 2585 DEGs with a threshold criterion of log FC greater than and equal to 2.5 and p-value less than 0.05 as the cut-off point were diagnosed. Among them, 1971 genes were upregulated and 615 genes were downregulated. The gene ontology resources showed fold enrichment pathways for up and downregulated genes (Figure 2 and Figure 3 respectively). The integrated pathways from KEGG database for differentially expressed genes comprise of Fat digestion and absorption, Cholesterol and Glycerolipid metabolism pathways.

The protein- protein interactions (PPI) between DEGs were derived from STRING database. The PPI network was represented in the form of nodes and edges, where each node represented a protein product of single-gene and edges represented the protein-protein association. The network backbone of identified upregulated genes comprised of 1816 nodes and 13477 edges with an estimated clustering coefficient 0.242 (Figure S1 - check with authors). Similarly, the network backbone of identified downregulated genes comprised of 481 nodes (Proteins) and 2922 edges (interactions) with an estimated clustering coefficient 0.404 (Figure S2 - check with authors).

**Figure 4:**
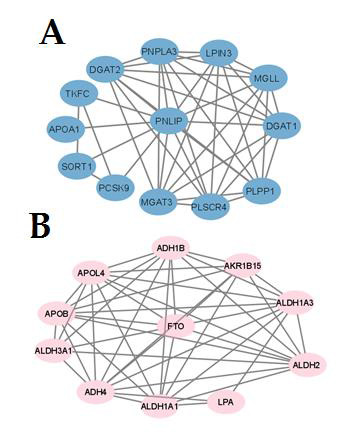
The Cluster 1(PNLIP) derived from the protein-protein interactions (PPI) network using MCODE with a score of 9.75 and 4b: Cluster 2 (FTO) derived from the protein-protein interactions (PPI) network using MCODE with a score of 8.04. Ellipse and lines represent the nodes and edges, respectively

After STRING analysis, Cytoscape was used to visualize and identify the PPI network. MCODE plugin (version 2.0.0) was used to identify the hub genes, and the parameters of DEG clustering and scoring were as follows: For cluster1 MCODE score=9.750, Degree Cut-off=2, Node Score Cut-off=0.2, k-score=2, and Max. Depth=100. It consists of 89 nodes and 429 edges. For Cluster2, MCODE score=8.048, Degree Cut-off=2, Node Score Cut-off=0.2, k-score=2, and Max. Depth=100. It consists of 126 nodes and 503 edges. The most densely interconnected regions of the protein -protein interactions (PPI) using MCODE for differentially expressed genes in Table 2. The first neighbour of PNLIP (Pancreatic Lipase) from cluster 1 and FTO (Fat mass and obesity protein) from cluster 2 was obtained and represented in Figure 4a and 4b respectively.

## Discussion:

The differentially expressed gene set showed both up and downregulated genes. Within, up-regulated gene set, the PNLIP (Pancreatic lipase) gene was the most significantly altered (log2FC=11.08); followed by FTO (Fat mass and obesity associated protein) log2FC=11.06. The high expression of PNLIP and FTO was reported for fat digestion and absorption cholesterol metabolism. Earlier studies reported that PNLIP [19] and FTO [20] genes were found to be potential targets for obesity. For the downregulated genes, PLCL1 (Lipase) with a log2FC of -7.85 was most significantly expressed followed by PDE4B (phosphodiesterase 4B) with log2FC of -7.82). PLCL1 was reported for fatty acid metabolism and PDE4B was reported for fat mass control and metabolic regulation. The functional enrichment analysis showed that genes such as AGPAT2, AKR1B10, AKR1B15, LDH1A1, ALDH1A3, ALDH2, ALDH3A1, ALDH7A1, ANGPTL4, APOA1, APOA4, APOB, APOBEC1, APOC1, APOC3, APOE, APOL4, CYP27A1, CYP2B6, CYP2B7P, CYP2C18, CYP2C19, CYP2C9, CYP2J2, CYP2S1, CYP2U1-AS1, DGAT1, DGKG, FABP1, GK, LPA, MGAT3, MGAT3-AS1, MGLL, PCSK9, PLPP1, PLSCR4, SOAT2, SORT1, TKFC were found in obesity related pathways. The fold enrichment analysis of upregulated gene includes pathways such as fat digestion and absorption pathway, glycerolipid metabolism, cholesterol metabolism, etc that are related to obesity. The above mentioned DEGs were found in these fold enrichment pathways related to obesity. The PPI interactions from Cluster 1 PNLIP, neighbourhood genes include PNPLA3, LPIN3, MGLL, DGAT1, PLPP1, PLSCR4, MGAT3, PCSK9, SORT1, APOA1, TKFC, DGAT2 genes whereas for cluster 2 FTO gene, first neighbour includes ADH1B, AKR1B15, ALDH1A3, ALDH2, LPA, ALDH1A1, ADH4, ALDH3A1, APOB, APOL4 genes.

## Conclusion:

Meta-analysis of 313 RNA seq dataset of 258 obese samples and 55 lean samples retrieved from GEO database includes six studies namely GSE152991, GSE132831, GSE86430, GSE148892, GSE161042 and GSE137631. The differential gene expression analysis showed 1970 genes were up regulated, and 615 genes were downregulated with a threshold criterion of log FC greater than and equal to 2.5 and p-value less than 0.05. The protein -protein interaction network analysis showed that PNLIP and FTO genes were identified as candidate genes targeting obesity.

## Figures and Tables

**Table 1:** Next generation RNA sequencing samples from GEO Database

**GEO Dataset**	**Sample type**
GSE152991	11 - Lean, 34 - Obese
GSE132831	224 - Obese
GSE132831	224 - Obese
GSE132831	224 - Obese
GSE132831	224 - Obese
GSE137631	6 - Lean

**Table 2:** The most densely interconnected regions of the protein -protein interactions (PPI) using MCODE for differentially expressed genes

**S. No.**	**MCODE score**	**Nodes**	**Edges**	**Nodes IDs**
1	9.75	89	429	APOC3, SERPINA1, LAMA1, CYP4F2, APOA1, C8A, LAMB2, RHOC, LAMB3, PIPOX, MT-ATP6, CLDN7, MLXIPL, MT-ND3, MT-ND4L, SLC51A, CLDN2, ADH1C, COL16A1, COL12A1, CTNND1, COL14A1, PARD3, SULT1C2, SFRP1, LAMC2, COL9A3, VTN, C6, KRT8,COL13A1, AOX1, HAAO, TJP3, MT-ND6, ALDH1A1, UGT1A1, MT-CO2, TM4SF4, COL25A1, COL27A1, P4HA2, G6PC, CYP4F3, ITGB4, AGXT, XPNPEP2, ALDOB, CDH17, HGD, LRP5, ALDH1A3, MT-ND2, CLDN12, MT-ND1, DKK1, ADH1B, ADH1A, WNT11, ITGA3, ITGB6, CLU, WNT5B, ALDH3A1, CLDN15, F12, CYP2J2, PTGS1, PLOD2, COL9A2, ITGB8, ADH6, MT-CO1, TMPRSS6, LPA, MT-CYB, CLDN11, LAMA3, ADH4, MT-ND4, F11, MT-ATP8, BCAR1, ITGB5, MTTP, CLDN1, CLDN23, LAMB1, FABP1
2	8.048	126	503	PPL, CSK2, CTTN, FOXA2, GABRE, PDX1, UGT1A10, NTS, MOGAT2, PPAP2C, NCKAP1, SLCO2B1, GAST, SDC2, PDLIM4, GHRL, AMBP, LPIN3, HMGCS2, GABRA4, OEBT, CLIC5, A1CF, UGT1A4, GSTA1, CYB5A, PLA2G4F, GABRB1, INSM1, LUM, GPC4, PLA2G12B, PNLIP, APOC1, KRT19, DKK3, CYP3A5, ANPEP, ESPN, ERBB3, GABRB3, EVPL, CEACAM5, CFTR, FZD5, UGT1A5, DGAT2, RBP1, FBLN1, MGST2, CD8A, PYY, GHRH, DGAT1, NPC1L1, ANG, PPARGC1A, SLC51B, GSTA4, FRZB, SST, PCSK1, BEST4, UCN3, FMOD, SLC10A2, SDC4, UGT1A3, ABCC8, CD24, NT5E, PPAP2B, EPHA2, ABCG5, PDLIM1, MGST3, GABRA2, PCK2, EFNA1, CCK, CREB3L3, TIMP3, CDX2, GSTA2, MET, NR0B2, EDN3, DSC2, DPYSL3, MYO1A, GC, MLN, MICALL2, CYP2C9, RFX6, ALOX12B, EPPK1, GAL, MYO1C, RBP4, IHH, PLA2G2A, EDN2, GABRP, MYO6, SLC2A2, GPX2, PCSK9, MOGAT3, TTR, EXOC3L4, CD9, GIP, KRT7, TBX3, SFRP2, PLA2G4A, MAFB, FTCD, APOA4, UGT2B17, PNPLA3, FABP2, HPGDS, PAX4, MGST1
